# Serum lipid profile patterns in patients with oral cancer and oral potentially malignant disorders

**DOI:** 10.6026/973206300220328

**Published:** 2026-01-31

**Authors:** Priyanka Singh, Sangeeta Gupta, Gaurav Gupta, Deepika Patidar, Dinesh Patidar, Ishita Gupta

**Affiliations:** 1 Department of Oral Medicine and Radiodiagnosis, K. M. Shah Dental College, Sumandeep Vidyapeeth University, Waghodiya, Vadodara, Gujarat, India; 2 Department of Physiology, All India Institute of Medical Sciences (AIIMS), Gorakhpur, Uttar Pradesh, India; 3 Department of General Surgery, All India Institute of Medical Sciences (AIIMS), Gorakhpur, Uttar Pradesh, India; 4 Department of Pedodontics, College of Dental Science and Hospital, Rau, Indore, Madhya Pradesh, India; 5 Department of Trauma and Emergency, All India Institute of Medical Sciences (AIIMS), Bhubaneswar, Odisha, India; 6 Government Institute of Medical Sciences, Noida, Uttar Pradesh, India

**Keywords:** Oral cancer, oral potentially malignant disorders, lipid, cholesterol, triglycerides, low density lipoprotein, high density lipoprotein and very low density lipoprotein

## Abstract

The role of serum lipids as tumour markers in the diagnosis of oral cancer and oral potentially malignant disorders (OPMDs) is of interest. Hence, three groups (oral cancer, OPMDs and age and sex matched healthy controls) comprising 40 patients each was selected. Serum lipid profile values (total cholesterol, triglycerides, low density lipoprotein, high density lipoprotein and very low density lipoprotein) were compared among the groups (unpaired t-test and one way-ANOVA) (p-value of <0.05 was considered as significant). Mean values of total serum cholesterol and low density lipoprotein were found to be elevated in cases of oral cancer and oral PMDs as compared to controls with statistical significance (p<0.001). The alterations in levels obtained in the study, support the role of serum lipid profile as biomarkers for oral cancer and OPMDs, yet strengthening of the evidences by long-term follow-up studies with larger sample sizes are warranted to establish the association.

## Background:

Oral cancer is one of the most common cancers in the world. Squamous cell carcinoma comprises about 90 to 95% of all oral malignancies and hence, the term 'oral cancer' is used in a restricted sense to describe squamous cell carcinoma [1]. Oral cancer is also considered to be preceded by various mucosal lesions known as oral potentially malignant disorders (OPMDs) for example Leukoplakia, Lichen Planus and Oral Submucous Fibrosis (OSMF) [2]. Biochemical studies in evaluation of cancer have shown that various substances alter quantitatively in the serum during tumour development and are referred to as tumour markers. So, if the biochemical changes occur even before frank cancer has occurred, we can predict even in oral precancerous lesions and conditions whether a particular individual is at risk or not [3]. The development of a tumour is largely contingent on uncontrolled and excessive cell growth. These rapidly proliferating cells require a number of essential components well above the normal limits required in physiological process. Lipids form one such essential component of cell membrane crucial for various biological functions including cell division and growth of normal and malignant tissues. Increased use of lipids by these rapidly dividing cells has been implicated in the reduction of the lipid stores [4]. The idea of screening such patients for the lipid profile is appealing owing to both the ease and the economic advantage of the investigations. Several researchers have reported the association of plasma/serum lipids and lipoproteins with different cancers and inverse relationship between serum lipid profile and OC and OPMDs as compared to age and sex matched healthy controls has been suggested [5, 6]. However, it is still debatable that the hypolipidemia in cancer is a cause or consequence despite considerable advancements in the cancer diagnostics approach. In addition, there are few reports of plasma lipid profile alterations in head and neck cancers in population from Western India. Therefore, it is of interest to assess the serum lipid profile in patients with oral cancer, OSMF, leukoplakia and lichen Planus in this region and analyse the alterations obtained, if any. Oral cancer is one of the most common cancers in the world. Squamous cell carcinoma comprises about 90 to 95% of all oral malignancies and hence, the term 'oral cancer' is used in a restricted sense to describe squamous cell carcinoma [1]. Oral cancer is also considered to be preceded by various mucosal lesions known as oral potentially malignant disorders (OPMDs) for example Leukoplakia, Lichen Planus and Oral Submucous Fibrosis (OSMF) [2]. Biochemical studies in evaluation of cancer have shown that various substances alter quantitatively in the serum during tumour development and are referred to as tumour markers. So, if the biochemical changes occur even before frank cancer has occurred, we can predict even in oral precancerous lesions and conditions whether a particular individual is at risk or not [3]. The development of a tumour is largely contingent on uncontrolled and excessive cell growth. These rapidly proliferating cells require a number of essential components well above the normal limits required in physiological process. Lipids form one such essential component of cell membrane crucial for various biological functions including cell division and growth of normal and malignant tissues. Increased use of lipids by these rapidly dividing cells has been implicated in the reduction of the lipid stores [4]. The idea of screening such patients for the lipid profile is appealing owing to both the ease and the economic advantage of the investigations. Several researchers have reported the association of plasma/serum lipids and lipoproteins with different cancers and inverse relationship between serum lipid profile and OC and OPMDs as compared to age and sex matched healthy controls has been suggested [5, 6]. However, it is still debatable that the hypolipidemia in cancer is a cause or consequence despite considerable advancements in the cancer diagnostics approach. In addition, there are few reports of plasma lipid profile alterations in head and neck cancers in population from Western India. Therefore, it is of interest to assess the serum lipid profile in patients with oral cancer, OSMF, leukoplakia and lichen Planus in this region and analyse the alterations obtained, if any.

## Materials and Methods:

A total of 120 subjects (age-range: 20 to 60 years) visiting the department of Oral Medicine and Radiology, Karnavati School of Dentistry and Hospital, Gandhinagar were included in the study. Obese patients, pregnant women, patients with family history of hyperlipidaemia, patients on medication for leukoplakia and OSMF and those with any systemic illness were not included. Sample size calculation was done based on the differences in the mean (effect size) from the previous similar study, power of 80% and 1.96 as the level of statistical significance [7]. The study was approved by the ethical committee of the institute. Written consent form was signed and obtained from the participants. The subjects were divided into the three groups. Group 1 consisted of 40 histopathologically diagnosed cases of oral cancer (OC). These patients were given TNM staging and histologically divided into well differentiated, moderately differentiated and poorly differentiated carcinomas. Group 2 consisted of 40 cases of oral potentially malignant disorders (18 cases of OSMF, 12 cases of leukoplakia and 10 cases of lichen planus). These lesions were biopsied and confirmed histopathologically except OSMF. Cases of OSMF were selected as per clinical diagnosis and divided into different clinical stages as per classification given by Khanna and Andrade [8]. Group 3 consisted of 40 subjects which was a control group of healthy subjects. Five ml of fasting venous blood was withdrawn from each subject. Analysis for TC, TG, HDL, LDL and VLDL) were carried out at the Qualitech Diagnostics, Ahmedabad, Gujarat, India by spectrophotometric method using fully automated chemistry analyzer, "Turbochem100. Data was tabulated and statistical analysis was performed using SPSS software version 20.0. P-value less than 0.05 was considered significant while p-value less than 0.001 was considered as highly significant. p<0.001 for alteration in cholesterol (CHL) and low density lipoprotein (LDL) levels (one-way ANOVA), while p>0.05 for triglyceride (TG), high density lipoprotein (HDL) and very low density lipoprotein (VLDL) level alterations.

## Results and Discussion:

**Figure 1:**
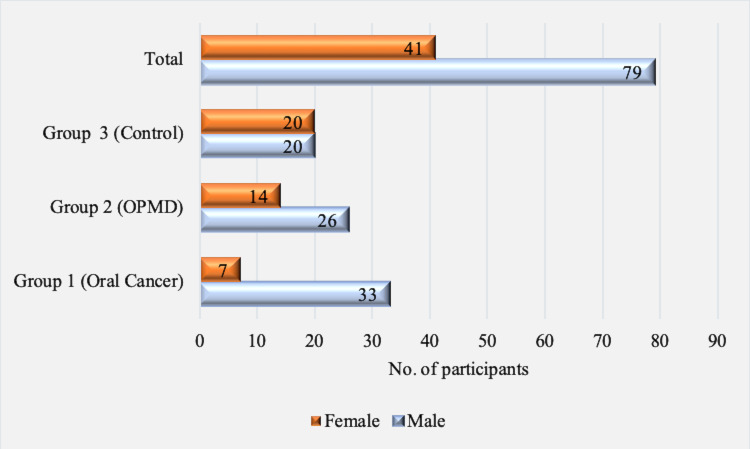
Gender distribution among all the three groups (Oral cancer, Oral potentially malignant disease (OPMD) and Controls)

**Figure 2:**
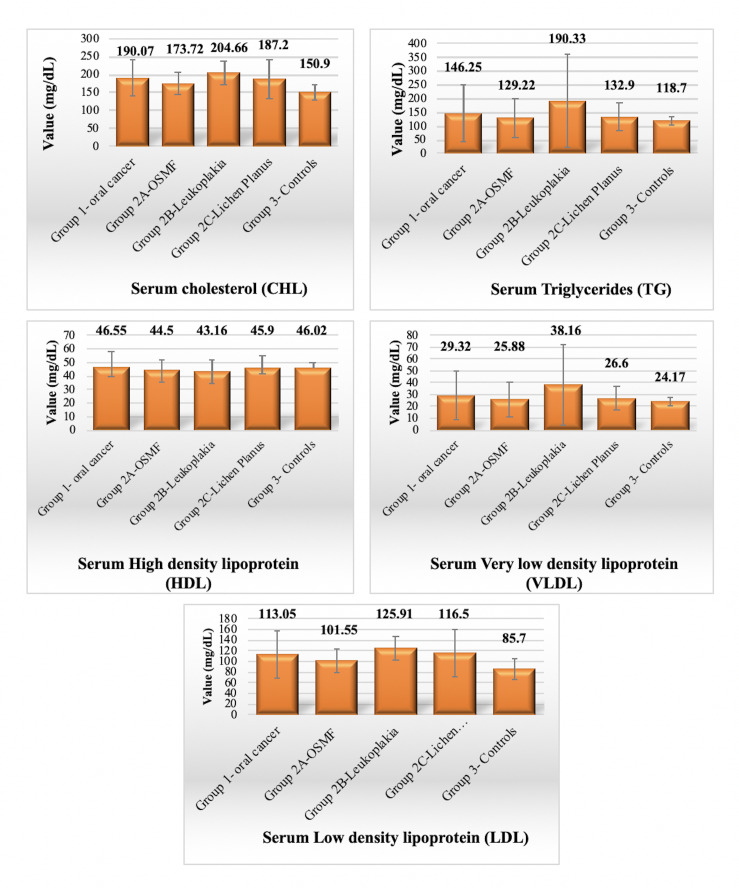
Comparison of lipid profile among all the groups with subdivisions

The three groups comprised 40 age-matched participants in each. Group 1 (oral cancer) had 26 males and 14 females; 33 males and 7 females constituted the group 2 (OPMD) and the healthy control group (group 3) had 20 males and 20 females (Figure 1). Majority of the subjects in the oral cancer group were having a habit of tobacco consumption. In the present study, the mean values of all lipid profile parameters (CHL, TG, LDL and VLDL) in Group 1 (oral cancer) and Group 2 (OPMD) were raised as compared to Group 3 (healthy subjects) (except lowered mean HDL levels in group 2). Statistical significance for the elevations obtained for CHL, TG, LDL and VLDL levels were found for CHL and LDL only (p<0.001) (one-way ANOVA) (Figure 2). Lowered mean values of HDL (mg/dL) in Group 2 (44.5 ± 7.03 in OSMF, 43.16 ± 9.14 in leukoplakia and 45.9 ± 9.19 in lichen planus as compared to controls (46.02 ± 4.07) is in line with the study done by Kamath *et al.* 2014 in which HDL was reported to be significantly decreased in cases as compared to controls [9]. The findings have been explained on the basis of the elevated lipid peroxidation in the cases of oral cancer and OPMD as tobacco carcinogens generate (reactive oxygen species) ROS and lipid peroxides, enhance lipid peroxidation and lead to tissue injury. Thus, lipid peroxidation has been suggested to play a role in low HDL in cancer patients. Their study was also characterized by marginal elevation of mean values of total cholesterol (TC), TG, LDL and VLDL. The raised values noted concord with the present study. Exposure to tobacco carcinogens, which hampers the antioxidants' defence, has been implicated in the elevation found [9]. A similar trend of alteration in the lipid profile was also reported by Alexopoulos *et al.* who identified 23 out of 100 patients demonstrating abnormally high values of serum total cholesterol/LDL/triglycerides/combination of above [10]. The present study also obtained increased levels of CHL, TG, VLDL, HDL and LDL in Group 1 (oral cancer) as compared to Group 2 (OPMD) but without statistical significance (P>0.05) (unpaired t-test) (Table 1). However, the present study findings contradict majority of the retrospective and prospective studies done in the past which have shown that lipid levels decline in oral squamous cell carcinomas [11, 12- 13]. Increased utilization of lipids for new membrane biogenesis by rapidly dividing cells in premalignancies and malignancies have been attributed as the underlying cause for the lowered levels. A plausible role of lipids as a metabolic marker in the etiopathogenesis, diagnosis and prognosis of OPMDs and cancer has been widely explored [14, 15]. Reprograming of lipid metabolism has been proposed which in turn enhances the oncogenic signalling via growth factor pathways leading to tumour proliferation and invasion. This has also gained attention as a new hallmark of cancer [16]. However, a metanalysis by Gupta *et al.* (2025) has concluded that the lowered lipid levels were significant for the comparison between OSMF patients and healthy controls, while no statistical significance could be derived for the comparison of controls with oral squamous cell carcinoma patients [17]. They emphasized on the heterogeneity of the studies. The methodological variations of studies in estimating lipid levels, the instruments and the analytical procedures used for measurement were attributed for the heterogeneity. Moreover, they highlighted that the lipid profile measured once could not represent a person's average lipid levels and cause within-person variations.

Alteration in the lipid profile albeit with significantly elevated levels of total cholesterol and LDL and marginal lowering of HDL levels obtained in our study can hence be attributed to the sample size variation, geographical variation (studies done in Punjab where people prefer high calorie diet and hence the mean values of lipid profile in healthy subjects remain higher), different age-ranges as well as different socio-economic status of the participants as major causes of incongruent results with respect to the previous similar studies. Choi *et al.* have suggested that the hypercholesterolemia could be due to decreased levels of serum antioxidative vitamins in the cancer patients. Following a decrease in the level of antioxidative vitamins in the serum, there is increase in the number of free radicals which then increases lipid peroxidation [18]. Notwithstanding, majority of the similar studies (including the present study) depicted a characteristic alteration in the lipid profiles in oral cancer and/or OPMD. Hence, assessment of the lipid profile in tobacco consumers/ OPMD/oral cancer patients might reflect alterations in the oral mucosa leading to the development of OPMDs or their malignant transformation. Also, the major bulk of studies reporting altered lipid levels support the proposed explanation regarding the role of rapidly dividing cells and enhanced utilization of lipids in malignancy.

## Conclusion:

We show significant alteration of total serum cholesterol and LDL levels in oral cancer and OPMD subjects. Impaired antioxidants' defence due to the exposure to tobacco carcinogens might have led to the elevation of lipids found. On the other hand, marginal lowering of HDL in OPMD subjects might be due to elevated lipid peroxidation in OPMDs. Considering the fact that the alteration in lipid levels is contingent upon the geographical variation, dietary habits, nutritional status and other physical activities, further long-term follow-up studies with larger sample sizes are warranted. Establishing the role of lipid profile as biomarkers in oral cancer and OPMD could aid in the risk assessment and hence the early detection of the conditions.

## Advancement to knowledge:

The study strengthens the evidences of significant alteration of lipid profile in oral cancer and OPMD subjects and its role as metabolic marker in the above mentioned conditions. The study has also highlighted the fact that alteration in lipid profile has substantial evidences, yet the results are incongruent in terms of elevation or lowering of the levels of serum lipids in different studies including the current research. This highlights the importance of many influencing variables such as geographic variations, age-ranges and socio-economic status in lipid profile alteration observed in the studies.

## Funding:

None

## Ethical approval:

The study protocol was approved by the Institute's Ethics Committee (Karnavati School of Dentistry Ethics Committee (KSDEC), Gandhinagar, Gujarat, India (dated 27.11.2015).

## Figures and Tables

**Table 1:** Comparison of lipid profile values between group 1 (oral cancer) and group 2 (oral potentially malignant disorders)

**Group**	**Number of participants**	**Value (mg/dL)**		**p value**
		**Mean**	**SD**	
**Cholesterol (CHL)**				
Group 1	40	190.07	50.4	0.716 NS
Group 2	40	186.37	39.48	
**Triglyceride (TG)**				
Group 1	40	146.25	103.67	0.925 NS
Group 2	40	148.47	108.22	
**High density lipoprotein (HDL)**				
Group 1	40	46.55	11.23	0.341 NS
Group 2	40	44.45	8.1	
**Very low density lipoprotein (VLDL)**				
Group 1	40	29.32	20.72	0.929 NS
Group 2	40	29.75	21.67	
**Low density lipoprotein (LDL)**				
Group 1	40	113.05	43.67	0.957 NS
Group 2	40	112.6	30.28	
p-value: >0.05 [NS (not significant)]
(unpaired t-test)
Group 1: Oral cancer and
group 2: Oral potentially malignant disorders
(oral submucous fibrosis,
leukoplakia and lichen planus).

## References

[1] Tan Y (2023). Int J Oral Sci..

[2] Lorini L (2021). Cancers (Basel)..

[3] Garg D (2014). J Int Oral Health..

[4] Sai SK (2022). J Oral Maxillofac Pathol..

[5] https://ijrar.org/papers/IJRAR2002127.pdf.

[6] Khyalia S (2022). Asian Pac J Cancer Biol..

[7] Acharya S (2016). Int J Oral Maxillofac Surg..

[8] Khanna JN, Andrade NN (1995). Int J Oral Maxillofac Surg..

[9] Kamath A (2014). J Cancer Res Ther..

[10] Alexopoulos CG (1987). Cancer..

[11] Sai SK (2022). J Oral Maxillofac Pathol..

[12] Sai SK (2024). Oral Maxillofac Pathol J..

[13] Sangle VA (2023). Oral Maxillofac Pathol J..

[14] Ma H (2025). BMC Oral Health..

[15] Sachdeva R (2020). Indian Journal of Dental Sciences..

[16] Dong S (2023). Ther Adv Med Oncol..

[17] Gupta S (2025). J Oral Pathol Med..

[18] Choi MA (1999). Cancer Lett..

